# The influence of patient positioning and immobilization equipment on MR image quality and image registration in radiation therapy

**DOI:** 10.1002/acm2.14162

**Published:** 2023-09-16

**Authors:** Atefeh Rostami, Mostafa Robatjazi, Seyed Alireza Javadinia, Nematullah Shomoossi, Ramin Shahraini

**Affiliations:** ^1^ Department of Medical Physics and Radiological Sciences Sabzevar University of Medical Sciences Sabzevar Iran; ^2^ Non‐Communicable Diseases Research Center Sabzevar University of Medical Sciences Sabzevar Iran; ^3^ School of Medicine Sabzevar University of Medical Sciences Sabzevar Iran; ^4^ Department of Radiology School of Medicine Sabzevar University of Medical Sciences Sabzevar Iran

**Keywords:** brain tumor, magnetic resonance imaging, three dimensional (3D) conformal radiation therapy

## Abstract

**Introduction:**

MRI is preferred for brain tumor assessment, while CT is used for radiotherapy simulation. This study evaluated immobilization equipment's impact on CT‐MRI registration accuracy and MR image quality in RT setup.

**Methods:**

We included CT and MR images from 11 patients with high‐grade glioma, all of whom were immobilized with a thermoplastic mask and headrest. T1‐ and T2‐weighted MR images were acquired using an MR head coil in a diagnostic setup (DS) and a body matrix coil in RT setup. To assess MR image quality, signal‐to‐noise ratio (SNR) and contrast‐to‐noise ratio (CNR) were considered in some dedicated regions of interest. We also evaluated the impact of immobilization equipment on CT‐MRI rigid registration using line profile and external contour methods.

**Results:**

The CNR and SNR reduction was in the RT setup of imaging. This was more evident in T1‐weighted images than in T2‐weighted ones. The SNR decreased by 14.91% and 12.09%, while CNR decreased by 25.12% and 20.15% in T1‐ and T2‐weighted images, respectively. The immobilization equipment in the RT setup decreased the mean error in rigid registration by 1.02 mm. The external contour method yielded Dice similarity coefficients (DSC) of 0.84 and 0.92 for CT‐DS MRI and CT‐RT MRI registration, respectively.

**Conclusion:**

The image quality reduction in the RT setup was due to the imaged region's anatomy and its position relative to the applied coil. Furthermore, optimizing the pulse sequence is crucial for MR imaging in RT applications. Although the use of immobilization equipment may decrease the image quality in the RT setup, it does not affect organ delineation, and the image quality is still satisfactory for this purpose. Also, the use of immobilization equipment in the RT setup has increased registration accuracy.

## 1 INTRODUCTION

Computed tomography (CT) is a commonly used imaging modality in radiation therapy (RT) treatment planning due to its high geometric precision and ability to provide electron density information for dose calculation in treatment planning software (TPS).^1^ However, the integration of imaging modalities such as magnetic resonance imaging (MRI) and positron emission tomography (PET) can improve the accuracy of tumor and organ at risk (OAR) delineation in RT planning.^2^, ^3^, ^4^

MRI is especially useful in RT planning due to its superior soft tissue contrast and ability to provide functional information about tissues. As a result, CT and MR images are often fused together in TPS to aid in organ delineation. MR images are used to contour target volumes and OARs, while CT images are used for dose calculation based on electron density. This approach allows for more accurate and detailed RT planning, which can improve patient outcomes.

Although MRI‐based radiotherapy (MR‐RT) has become one of the major areas of interest in radiotherapy in recent years, there are some limitations to its use. The reproducibility of patient positioning during RT fractions is a critical step in radiation therapy.^5^ Immobilization equipment is often used during simulation and RT fractions to address this issue. Thermoplastic masks are considered the gold standard for brain and head‐and‐neck radiotherapy as they prevent head motion during treatment and ensure consistent patient positioning.^6^ However, this equipment is typically attached to underlying devices that are not compatible with the receiving coils used for diagnostic head and neck MRI. The rigid volume of the dedicated coils for head and neck imaging makes it difficult to set up the procedure and obtain high‐quality images for RT simulation purposes. Several studies have shown that immobilization equipment used during MRI imaging can improve positioning accuracy in the radiotherapy process, but it may also cause a decrease in image quality.^7^, ^8^ Furthermore, acquiring MRI images in the treatment position that require the use of immobilization equipment can enhance the accuracy of rigid registration with CT images for use in RT treatment planning, as demonstrated by multiple studies.^9^, ^10^ However, using flexible coils instead of dedicated diagnostic head/neck coils to accommodate immobilization masks may lead to a compromise in image quality. Kaza et al. studied the effect of using some sort of flexible coils against the dedicated head and neck coil on some parameters of image quality for brain and head and neck radiotherapy simulations. By considering different parameters, they concluded that a flexible coil (UltraFlexLarge18 in their study) was the selected one compared to others.^11^ Similarly, Wong et al. also evaluated the effect of using flexible coil instead of a dedicated one using American College of Radiology (ACR) phantom. The results of this study also showed that the image quality was acceptable in radiation therapy setup imaging using the flexible coils.^12^ However, the immobilization equipment was not applied in this study.

With the emergence of MRI‐guided radiation therapy systems, the workflow for patient positioning, immobilization devices, and image quality require further assessment. Therefore, this study aimed to evaluate the impact of a specially designed fixation base and thermoplastic mask on image quality of MR‐simulation for 11 patients with brain tumors who were referred to the radiotherapy department for RT. The effect of immobilization equipment on the accuracy of CT‐MRI image registration is also a key objective of this study. The novelty of this study was the specifically investigation of the effect of immobilization equipment on MR image quality conjunction with the image registration by quantitative analysis.

## 2 METHODS AND MATERIALS

### 2.1 Patients

CT and MR images of 11 patients (five men and six women) with brain high grade glioma were examined in this study. While a larger sample size could potentially strengthen the statistical power and generalizability of our findings, due to the resource limitations and need for careful coordination with treatment schedules beside the time constraints were limited the sample size of the study. Majority of the tumors were extra‐axially located in brain with an average planning target volume (PTV) of 192.42 ± 78 cc. For all of the patients, a thermoplastic mask and headrest with its dedicated positioning wedge were used in the positioning and fixation procedure. The average age and weight of the patients were 53 years and 72 kg, respectively. It is worth mentioning that, informed consent was obtained from all participants prior to their inclusion in the study. A detailed consent form was provided to each patient, outlining the study's purpose, procedures. Patients were given sufficient time to review the form, ask questions, and seek clarification before voluntarily providing their written consent. The signed consent forms were securely stored in accordance with institutional guidelines to ensure patient confidentiality and compliance with ethical standards.

### 2.2 CT imaging

CT images of 11 patients in the treatment position were acquired at 90 kVp and 110 mAs, ∼180 slices, 512 × 512 matrix size, with a 3‐mm thickness without an interslice gap in the transverse plane using a Siemens 16 slice SOMATOM Emotion CT scanner (Erlangen, Germany).

### 2.3 MR imaging

**FIGURE 1:**
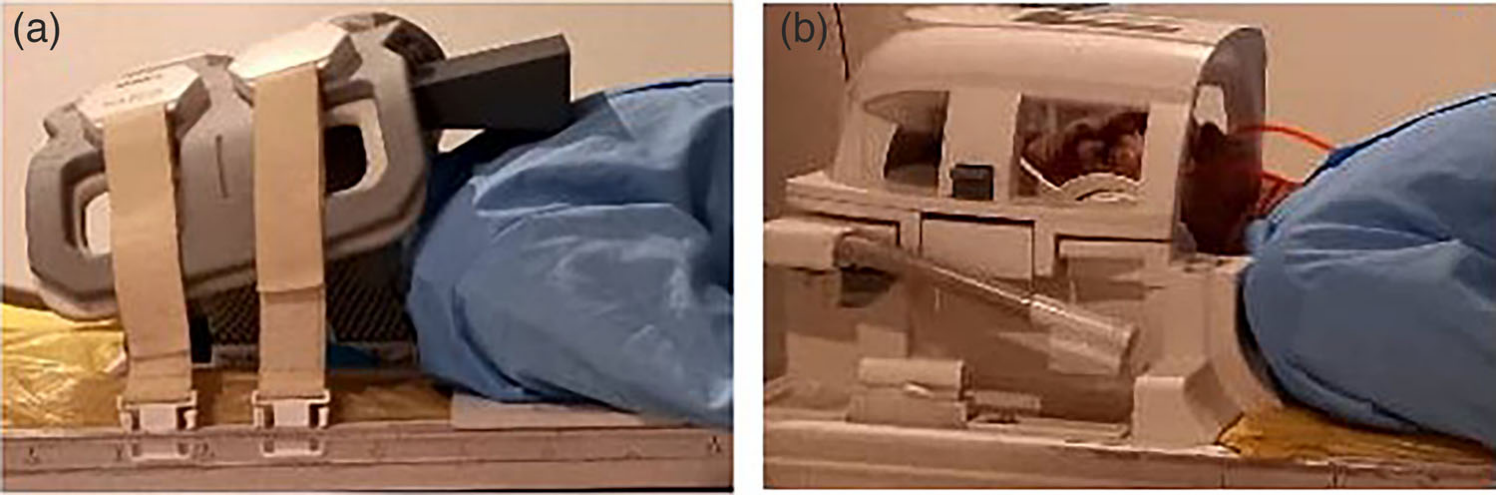
(a) RT setup by using fixing devices (thermoplastic mask, fixing base, and headrest) and 6‐channel body matrix coil, (b) DS setup with a head coil.

The MR image acquisitions were performed with a 55 cm bore Multive‐1.5T MRI scanner from Philips company (Philips Healthcare, the Netherlands). Patients were scanned using the standard T1 spine echo and T2 fast spin echo sequences with a head coil (diagnostic setup [DS]) and with 6‐channel body matrix coil directly strapped on the thermoplastic mask of the patient (radiotherapy setup [RT]). These setups are shown in Figure 1. The details of acquired T1 and T2 protocols are summarized in Table 1. To ensure that the pulse sequences did not affect image quality, it is important to note that we used similar pulse sequences for both the RT and DS setups.

**TABLE 1:** Details of applied pulse sequences.

Parameters	Protocols	
Pulse Sequence description	T1‐SE	T2‐FSE
	T1‐spin echo	T2‐Fast spin echo (FSE)
MR acquisition type	2D	2D
Slice thickness	5 mm	5 mm
Repetition time	279 ms	8048 ms
Echo time	6 ms	100 ms
Spacing between slices	0 mm	0 mm
Pixel bandwidth	534 Hz	111 Hz
Flip angle	90°	90°

### 2.4 Evaluation of MR image quality

The evaluation of MR image quality was performed patient‐by‐patient using image comparison between MR images in RT and DS setups. To achieve this goal, we calculated the signal‐to‐noise ratios (SNR) and contrast‐to‐noise ratios (CNR) of T1 and T2 weighted MR images in six specific regions of interest (ROIs). These ROIs were manually identified in the middle slice of the brain tissue, where the lateral ventricles are visible in the images. This approach eliminated the effects of ROI placement on image quality variations. The six ROIs include the right lateral, left lateral, anterior, posterior, center, and background regions of the image. The circular ROIs were considered 5 cm^2^ in the brain tissue and background in both MRI images of DS and RT setups. Delineation and quantification of ROIs in the images were done by using Segment Editor and Segment Quantification modules of 3D slicer software (version 4.10).

**FIGURE 2:**
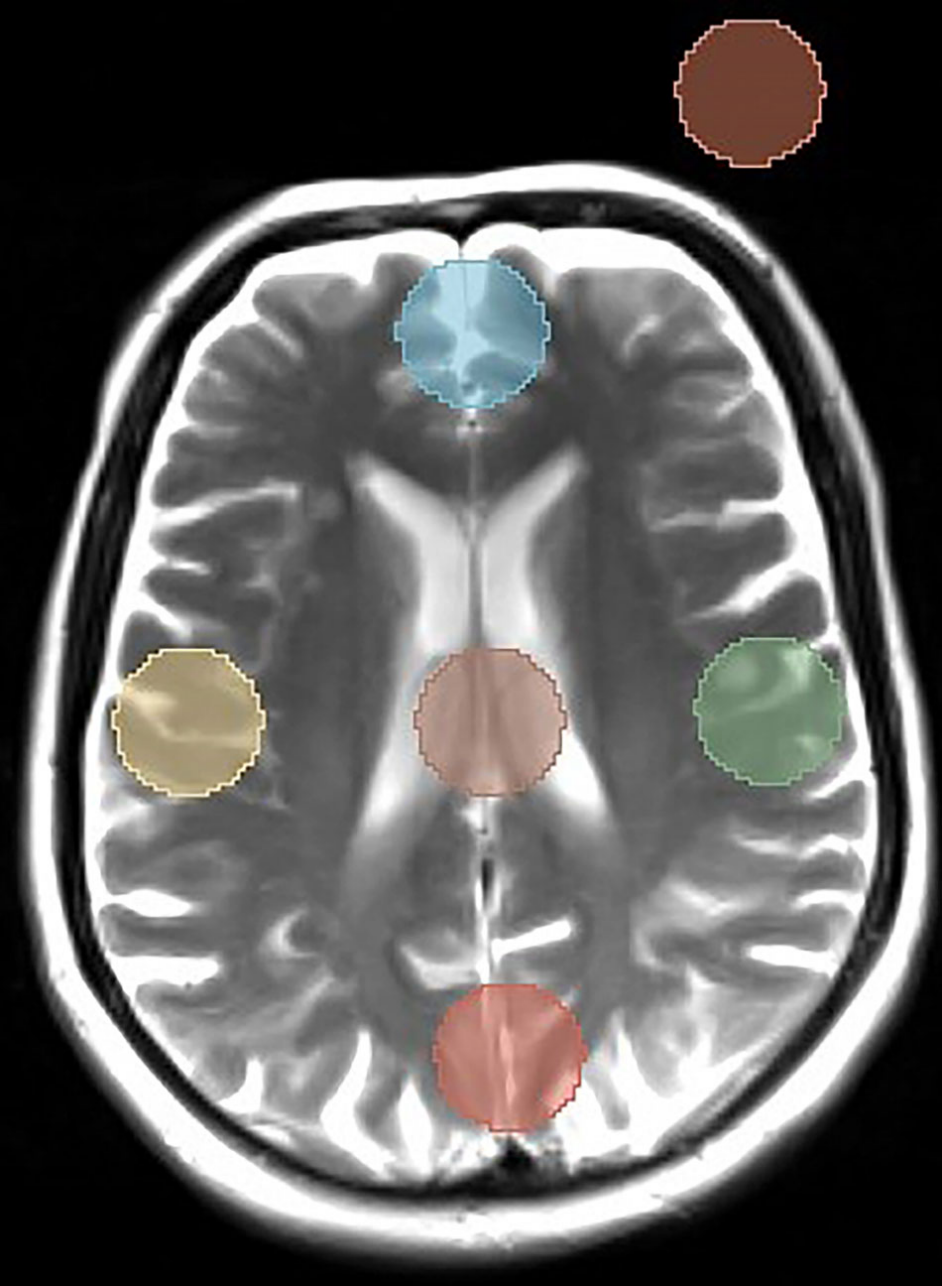
Delineation of six ROIs for SNR calculation of MRI images in RT and DS setups.

Figure 2 shows the selected regions for the evaluation of image quality using SNR and CNR in MR images. For each image, the average signal intensity was calculated based on Equation (1) which is described as follows; right, left, superior, inferior, and center ROIs, and the amount of noise was considered as the standard deviation (SD) of the signal intensity in a background region.^13^

(1) $$SNR=\frac{\mathrm{Average}\;\mathrm{Signal}\;\mathrm{Intensity}\;\mathrm{of}\;\mathrm{Selected}\;\mathrm{Region}}{\mathrm{Standard}\;\mathrm{Deviation}\;(\mathrm{SD})\;\mathrm{of}\;\mathrm{the}\;\mathrm{Signal}\;\mathrm{Intensity}\;\mathrm{in}\;\mathrm{the}\;\mathrm{Backgound}}$$

For calculation of CNR values for each ROI, average values of the considered ROI and the background one was used in Equation (2) as follow:

(2) $$CNR=\frac{\mathrm{Average}\;\mathrm{Signal}\;\mathrm{Intensity}\;\mathrm{of}\;\mathrm{ROI}-\mathrm{Average}\;\mathrm{Signal}\;\mathrm{Intensity}\;\mathrm{of}\;\mathrm{Backgound}}{\mathrm{Standard}\;\mathrm{Deviation}\;(\mathrm{SD})\;\mathrm{of}\;\mathrm{the}\;\mathrm{Signal}\;\mathrm{Intensity}\;\mathrm{in}\;\mathrm{the}\;\mathrm{Backgound}}$$

### 2.5 Evaluation of the MR‐CT rigid registration accuracy

The effect of position correction on the MR‐CT image registration accuracy was evaluated by the line profile and external contour methods. The description of applied methods in the evaluation of image registration is presented below.

#### 2.5.1 The line profile method

The evaluation of image registration by this method relies on the fact that cortical bone has the maximum pixel values on CT images while it has minimum pixel values on MR images. Two sets of MR images (DS and RT setups) were registered on the reference CT images with the rigid registration method using the registration and transform modules of 3D slicer software. After registration, a line was considered in the central slice of the brain and the pixel values of the line profile were acquired from CT and T2 weighted MR images in DS and RT setup. Because of the superiority of T2 weighted MR image quality, these MR images were selected for the evaluation of image registration accuracy.

**FIGURE 3:**
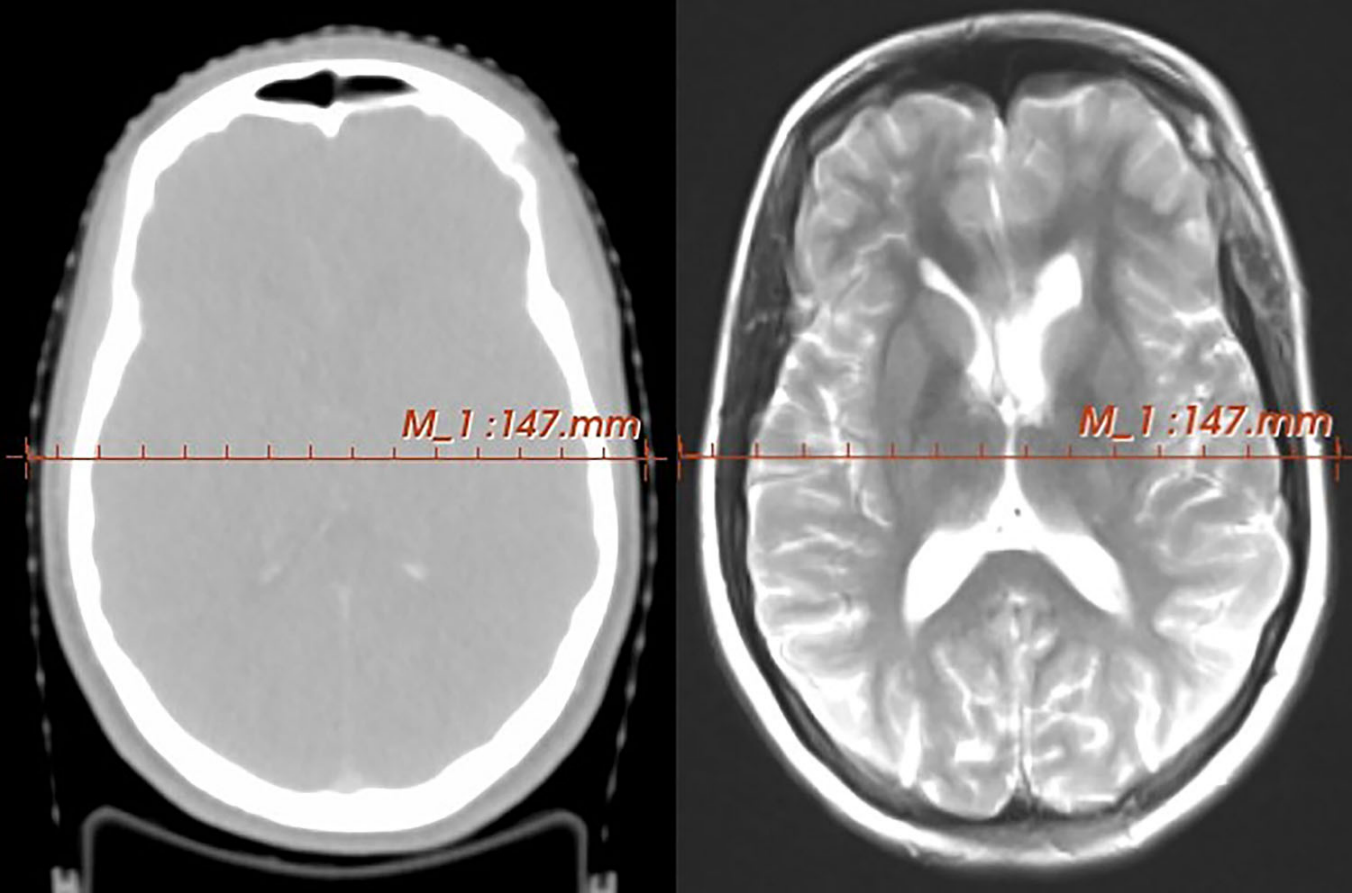
Considered lines for pixel values evaluation in CT and MR images.

Line profile quantification was performed by the LineProfile module of 3D slicer software. The difference between the place of the maximum pixel value in CT images and the minimum pixel value in MR images was considered as a quantity for the evaluation of image registration accuracy. It is worth mentioning that, after the registration process, both imaging datasets are considered as a single entity as illustrated in the Figure 3, and the line profiles are obtained from this combined dataset. This ensures that the spatial coordinates between the two different imaging modalities are aligned accurately, thereby representing the effectiveness of the registration method described above.

#### 2.5.2 The external contours method

In this method, external contours of applied images in the registration process were acquired using threshold‐based segmentation. For this purpose, the Segment Editor module of the 3D slicer software was used. The threshold value for the external contours of the CT image was selected as −150 while this value for MR images was 40. Furthermore, since the pixel values can vary between patients, we also considered a 10% variation in the selecting thresholds. The segmented regions in the images were converted to binary label maps, and then the similarity of the contours was evaluated by the Dicecomputation module of 3D slicer software. The Dicecomputation module calculates the Dice similarity coefficient that quantifies the overlapping between two label maps via Equation (3):

(3) $$DSC=\frac{[2\times (\mathrm{CT}\;\mathrm{label}\;\mathrm{map}\cap \mathrm{MR}\;\mathrm{label}\;\mathrm{map})]}{\mathrm{CT}\;\mathrm{label}\cup \mathrm{MR}\;\mathrm{label}\;\mathrm{map}}$$

DSC has a restricted range from 0 to 1, and values closer to 1 indicate more accurate CT‐MR image registration.

## 3 RESULTS

### 3.1 Evaluation of images quality

**FIGURE 4:**
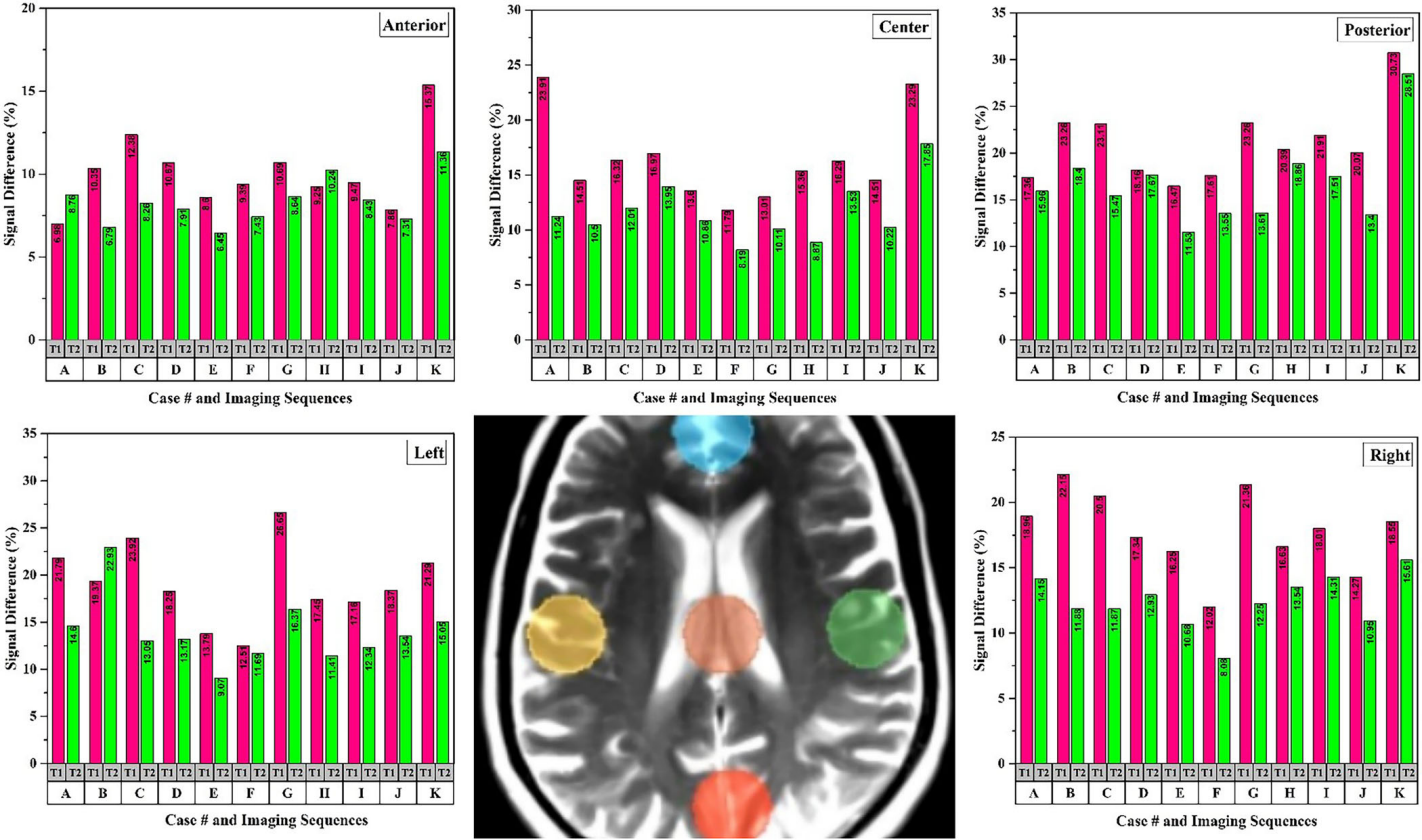
The figure demonstrates the signal difference in percentage between DS and RT setups for the considered ROIs in both T1 weighted (pink color bar) and T2 weighted (bright green color) images.

**FIGURE 5:**
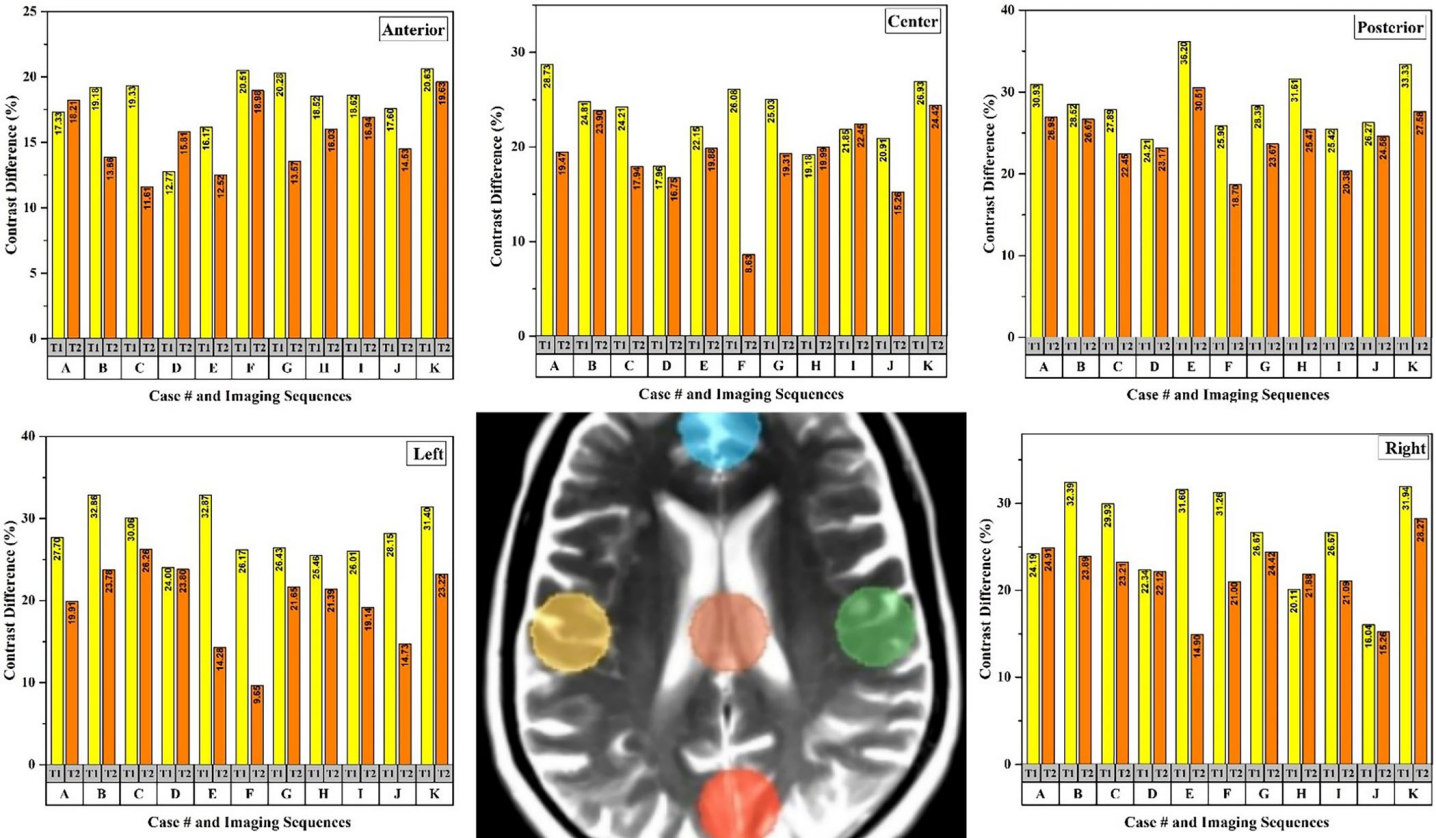
The figure demonstrates the contrast difference in percentage between DS and RT setups for the considered ROIs in both T1 weighted (yellow color bar) and T2 weighted (orange color) images.

**FIGURE 6:**
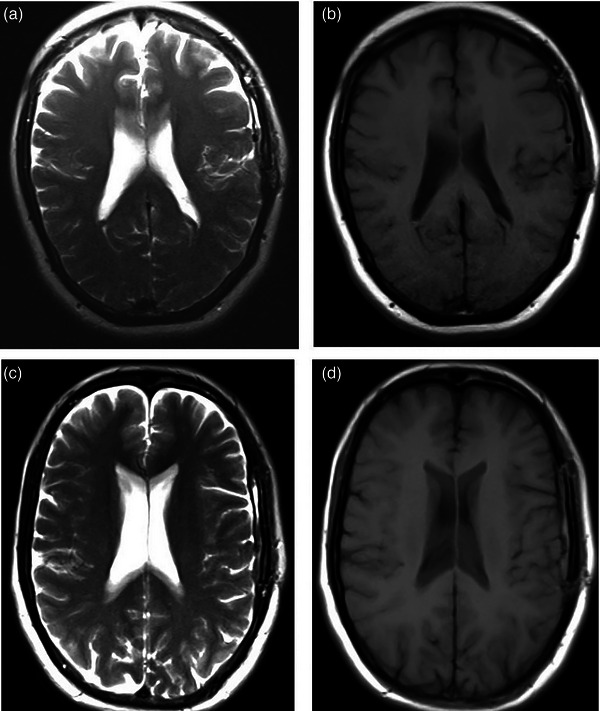
(a) T2 weighted and (b) T1 weighted MRI in RT setup; (c) T2 weighted, and (d) T1 weighted MRI in DS setup.

The SNR and CNR values for the considered regions on T1 and T2 weighted images in both DS and RT imaging setups were calculated. To assess the effects of positioning equipment and immobilization devices on the image quality, relative SNR and CNR reduction were calculated and shown in Figures 4 and 5. T2 and T1 weighted MR images of one patient in RT and DS setups have been shown in Figure 6.

As shown in Figure 4, the application of positioning equipment and immobilization devices in MRI led to a decrement in the SNR values. The maximum SNR reduction was seen in the patient with applying a wedge under the headrest for the flexed neck position, 30.73% and 28.51% for T1 weighted and T2 weighted images, respectively. One sample of T1 and T2 weighted images of the patient in flexed neck position in DS and RT setups is shown in Figure 5. The reduction of image quality is obvious in the RT position.

The average value of SNR and CNR changes and relevant deviations in two setups of MR imaging is shown in Table 2.

**TABLE 2:** The average values of SNR and CNRs reduction and relevant standard deviations of DS and RT setups.

	ROI					
Image quality parameter	Imaging sequence	Left	Right	Anterior	Posterior	Center
**SNR (%)**	*T*_1_	17.21 ± 4.08	16.14 ± 3.00	8.69 ± 1.47	18.33 ± 2.51	14.21 ± 3.16
	*T*_2_	13.82 ± 3.55	12.06 ± 1.18	8.02 ± 1.05	15.59 ± 2.37	10.95 ± 1.74
**CNR (%)**	*T*_1_	28.28 ± 2.93	26.64 ± 5.19	18.26 ± 2.19	28.97 ± 3.51	23.44 ± 3.17
	*T*_2_	19.80 ± 4.77	21.90 ± 3.76	15.60 ± 2.52	24.55 ± 3.22	18.91 4.21

### 3.2 Evaluation of image registration

**FIGURE 7:**
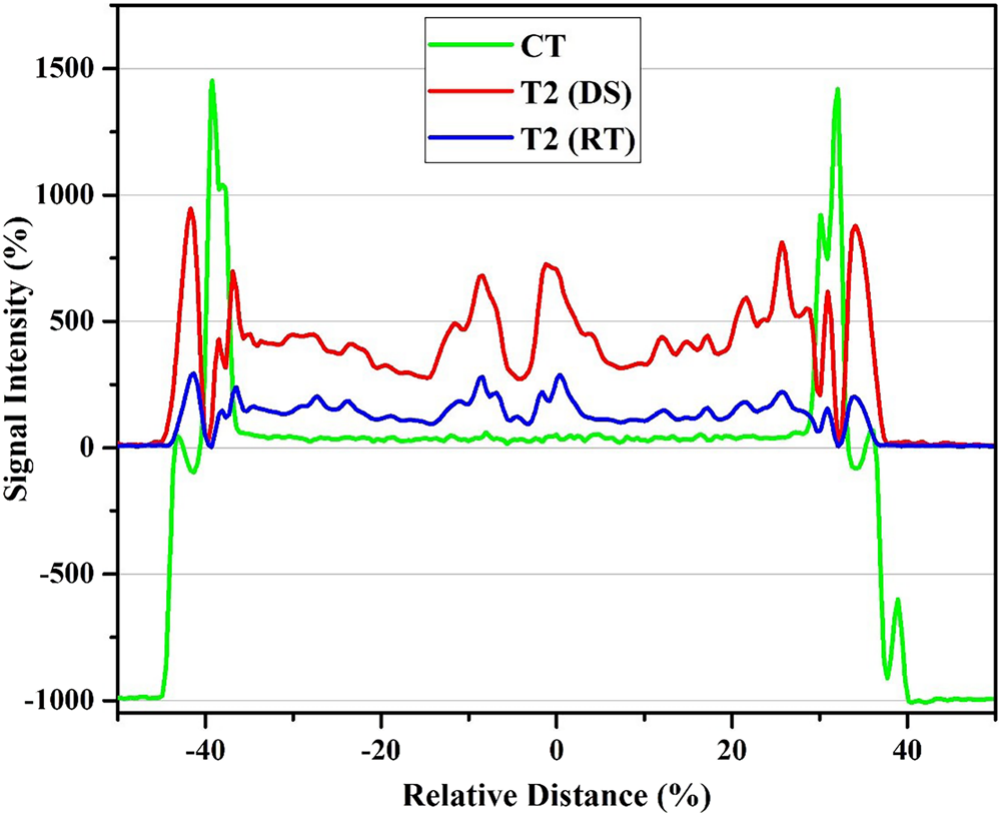
Line profiles of registered CT, T2 weighted MRI in DS, and T2 weighted MRI in RT setups (T2p: T2 weighted MRI with overlay plate in RT position). Due to combination of coordination in both imaging modalities, the illustrated relative distances are related to a common coordination.

Sample line profiles of registered CT, T2 weighted MRI in DS, and T2 weighted MRI in RT setups for a patient are shown in Figure 7. To evaluate the CT‐MR registration, the positions of maximum and minimum pixel values in CT and MR images reported are compared and presented in Table 3.

**TABLE 3:** The positions of maximum and minimum pixel values in CT and T2 weighted MR images in DS and RT position.

Patient	CT (left) max (mm)	CT (right) max (mm)	T2w‐DS‐left min (mm)	T2w‐DS‐right min (mm)	T2w‐RT‐left min (mm)	T2‐RT‐right min (mm)
1	20.55	141.84	24.38	147.20	21.38	140.51
2	19.10	145.74	16.34	148.86	18.38	146.86
3	24.91	160.14	21.11	155.15	23.05	159.11
4	24.34	139.56	21.02	136.15	22.45	139.02
5	26.82	157.32	30.60	152.26	26.16	158.58
6	20.43	141.73	16.15	145.00	22.26	142.28
7	37.77	170.14	41.02	173.38	37.59	172.42
8	27.07	146.57	23.66	151.10	25.57	147.09
9	21.64	145.94	18.39	141.05	22.59	146.84
10	21.41	149.32	24.26	144.63	21.24	149.53
11	23.04	160.17	18.23	166.23	21.11	162.05

The differences between the position of the maximum pixel value in CT images and minimum pixel values in DS and RT MR images based on the information of line profiles are reported in Table 4.

**TABLE 4:** The difference between the positions of maximum and minimum pixel values in CT and T2 weight MR images in DS and RT position.

Patient	CT (left) with T2w DS MRI (mm)	CT (right) with T2w DS MRI (mm)	CT (left) with T2w RT MRI (mm)	CT (right) with T2w RT MRI (mm)
1	3.84	5.36	0.17	0.33
2	2.76	3.12	0.73	1.13
3	3.80	4.99	1.86	1.02
4	3.32	3.41	1.89	0.54
5	3.79	5.06	0.66	1.26
6	3.28	4.28	1.83	0.55
7	3.26	3.24	0.18	2.28
8	3.40	4.53	1.50	0.52
9	3.25	4.90	0.95	0.90
10	2.85	4.70	0.17	0.21
11	4.81	6.06	1.94	1.87
**Average ± SD**	**3.49 ± 0.54**	**4.51 ± 0.89**	**1.08 ± 0.71**	**0.96 ± 0.62**

Results of the image registration evaluation based on the similarity of external contours of CT and DS and RT MRI images are reported in Table 5.

**TABLE 5:** Dice similarity coefficient (DSC) of external contours of registered CT, DS MR, and RT MR images.

	Rigid registration	
Patient	DSC CT‐DS MR	DSC CT‐RT MR
1	0.88	0.95
2	0.85	0.95
3	0.79	0.90
4	0.84	0.91
5	0.89	0.95
6	0.88	0.93
7	0.80	0.90
8	0.87	0.93
9	0.82	0.89
10	0.85	0.93
11	0.77	0.90
**Average ± SD**	**0.84 ± 0.04**	**0.92 ± 0.02**

## 4 DISCUSSION

There are several important aspects in application of MRI in RT including geometric distortion of MR images, correcting of patient positioning similar to treatment position, and pseudo‐CT construction for dose calculation. In this study, we employed MR images for the purpose of brain RT planning, as well as for the correction of patient positioning from DS imaging setup to RT setup during MR imaging.

The use of immobilization equipment in the RT setup of MR imaging, along with additional equipment such as a base plate and head‐rest wedge, can result in an increased distance between the coils and the body part being imaged. This can lead to a reduction in the signal received by the coil, ultimately affecting the image quality parameters including the SNR and CNR. Conversely, due to the geometrical limitations of the dedicated head coils, the use of flexible coils can be useful in the RT setup of MR imaging with immobilization equipment. This is because flexible coils can better conform to the shape of the immobilization equipment and the patient's anatomy, resulting in improved signal reception because of reduction in the distance between the body part and coils element. However, limited receive channels of the flexible coils can be an issue in the using of these coils during MR imaging in RT setup that can lead to sub‐optimal image quality (reduction of SNR and CNR).^14^, ^15^ The results of our study including reported values for CNR and SNR confirmed the above‐mentioned facts. As illustrated in Figures 4 and 5, both CNR and SNR reduced in RT setup in different regions. Because of shorter distance of the flexible coil from the patient's head compared to the other regions, the SNR and CNR reduction was less than other ROIs, while the maximum reduction of these values was seen on the posterior ROIs which can confirm the effect of coil distance on image quality. It was recommended by some studies that the lower image quality in the posterior part of the head can be improved by adding a small coil in the back of the base plate.^16^ By considering the mean values of SNR and CNRs from the five areas of anterior, posterior, central, left lateral, and right lateral, the SNR reduced by 14.91% and 12.09% and the CNR reduced by 25.12% and 20.15% in T1 and T2 weighted MR images, respectively. Taking into account these values, the reduction in SNR and CNR was greater in T1‐weighted than T2‐weighted images. Comparison of the image quality of MR images that acquired with different pulse sequences can be influenced by various intrinsic parameters of the pulse sequences. The fast spin echo sequences (T2) employ multiple 180° rephasing pulses, which can lead to an increase in SNR and, consequently, an increase in CNR. In contrast, the pulse sequence used for T1 images was a spin echo, resulting in lower SNR and CNR values compared to the fast spin echo sequence, because of lacking the multiple rephasing pulses.

The effect of immobilization devices of RT positions on the quality of MR images has been investigated in several studies. Winter et al. compared the SNR and CNR of PET‐MR images of ten patients with head‐and‐neck cancer in RT and DS setups. To facilitate the RT setup, a flat MR tabletop, an in‐house add‐on layer for patient immobilization with a thermoplastic mask, a pair of C‐shaped coil holders, and a 6‐channel flexible body matrix coil were used. However, the use of immobilization equipment in this study resulted in a reduction of SNR by approximately 26.2%.^17^ Similarly, Sun et al. conducted a study to evaluate the MR image quality of diagnostic DS and RT setup in the pelvic region, using a sample of 10 volunteers. Their findings revealed a reduction in SNR of approximately 27%−32.9% due to the use of an overlay table and a coil holder. Additionally, the CNR was reduced by approximately 24.4% in their study. However, despite the reduction in image quality in the RT setup, no significant effect was observed on prostate delineation.^7^ Xing et al. assessed the MR image quality during the installation and commissioning process of a dedicated 3T MR simulator. Their findings indicated that the use of a RT flat overlay table resulted in an average reduction of 42% of SNR in MR images.^8^ Our study reported a lower SNR reduction compared to the findings of Winter et al., Sun et al., and Xing et al. This difference could be attributed to the absence of an overlay flat table and coil holder in our study. Despite the observed reduction in SNR and CNR in RT MRI images compared to DS MRI images, the image quality appears to be adequate for RT planning. This is supported by several studies that have demonstrated good agreement between target structures delineated on MRI images in RT and DS setups.^7^, ^18^, ^19^

The second objective of our study was the evaluation of MRI image registration with the CT scan in the application of immobilization equipment during MR imaging. The mean error for rigid registration which was reported in Table 4 showed the improving the registration accuracy (77.38% decrement of the error) so that the mean error value was changed from 4.51 to 1.02 mm in the use of RT MR images than the DS MR images. The reported data in Table 5 also confirmed the effect of immobilization equipment during MR imaging. The mean DSC value in CT and RT MR images registration was about 0.92, while this value for the DS MR images was about 0.84. The DSC value was improved about 9.52% in the use of RT MR images.

Fortunati et al. conducted a comparison study on the registration of CT and MRI images for 10 patients with head and neck (H&N) tumors. They considered cases with and without immobilization equipment. Their findings demonstrated that employing deformable registration and positioning tools considerably enhanced registration precision.^9^ In an effort to demonstrate the impact of patient positioning on MRI registration accuracy, Hanvey et al. conducted a study involving 22 patients diagnosed with oropharyngeal cancer. The results of their study revealed that when patients were positioned in RT set up for MR imaging, there was a significant enhancement in the accuracy of rigid CT‐MR image registration, target delineation, and dose calculation.^10^ Building on the findings of these studies by Fortunati et al. and Hanvey et al. which was according with our study results, it can be inferred that implementing an immobilization device similar to those used in CT simulation for MRI imaging can effectively correct patient positioning and further improve the precision of CT and MR registration for RT purposes.

The small sample size in the present study can be considered the limitation of the study. Since MR imaging in two different positions (DS and RT setups) is tedious and time‐consuming, the cooperation of the patients was limited. On the other hand, more patients with different immobilization equipment including the prone position or using various wedges and head rests for the flexion and extension of the neck can increase the necessary information in the field of image quality and the accuracy of image registration with and without RT immobilization equipment. Another limitation of our study is that we evaluated and compared the image quality using 2D images. This was due to the fact that 3D image quality assessment can be time‐consuming and labor‐intensive, particularly when assessing multiple planes or volumes. However, 2D image quality assessment can be affected by variations in the image plane and orientation, which can lead to inconsistencies in the assessment. In contrast, 3D assessment is not limited by image plane or orientation, and can provide a more consistent and reliable assessment. Although we have incorporated CT images with a slice thickness of 3 mm and MRI images with a slice thickness of 5 mm, which have the potential to impact both objectives of the study, the results of the registration and image quality parameters have shown acceptable outcomes. It is important to consider that a thinner slice thickness can improve organ and target delineation, as well as enhance the accuracy of the registration process. However, reducing the slice thickness can also result in a decrease in SNR and CNR, which can ultimately affect the primary purpose of utilizing these images. Therefore, it is crucial for the radiation therapy team to strike a balance between slice thickness and image quality, taking into account the trade‐off involved.

## 5 CONCLUSION

We evaluated the effects of using a thermoplastic mask as immobilization equipment and the head rest on the MRI image quality and the accuracy of CT‐MRI image registration in the brain region. Our results showed that decreased image quality depends on the anatomical regions in the RT position. The maximum SNR and CNR decrement were observed in the posterior region of the MR images, because of distance from the coil elements. Although use of immobilization equipment reduced the image quality during MR imaging in RT setup, it did not affect organs delineation and image quality acceptable for this purpose. Also, the error of CT‐MRI image rigid registration decreased to about 1 mm in the RT setup of MR imaging.

## AUTHOR CONTRIBUTION

Study concept and design: **Atefeh Rostami** and **Mostafa Robatjazi**; acquisition of data: **Atefeh Rostami**, **Ramin Shahraini,** and **Seyed Alireza Javadinia**; analysis and interpretation of data: **Atefeh Rostami** and **Nematullah Shomoossi**; drafting of the manuscript: **Atefeh Rostami**, **Mostafa Robatjazi**; critical revision of the manuscript for important intellectual content: **Seyed Alireza Javadinia** and **Nematullah Shomoossi**; statistical analysis: statistician

## CONFLICT OF INTEREST

The authors report no conflicts of interest.

## ETHICAL APPROVAL STATEMENT

The protocol of the study was approved by the Ethics Committee of Sabzevar University of Medical Sciences (IR.MEDSAB.REC.1399.117). This research was carried out in line with the Helsinki Declaration. Since the data were collected anonymously and there were no interventions, obtaining a written informed consent form was waived by the Institutional Review Board of the Sabzevar University of Medical Science, Tehran, Iran.

## Data Availability

All data generated and analyzed during this study can be accessed through direct communication with the corresponding author and the agreement of all research team members.
